# Discriminating single-amino acid differences and post-translational modifications in negatively charged peptides with α-hemolysin nanopores

**DOI:** 10.1186/s12951-026-04818-z

**Published:** 2026-07-21

**Authors:** Liting Kang, Linting Wang, Xuefei Fan, Lingfang Xu, Han Yang, Chenxin Wu, Tongxin Zhu, Xiangyi Zhao, Linming Zhang, Jianhuang Xue, Shuanghong Yan

**Affiliations:** 1 Key Laboratory of Spine and Spinal Cord Injury Repair and Regeneration of Ministry of Education,, Tongji Hospital affiliated to Tongji University, Frontier Science Center for Stem Cell Research, School of Life Sciences and Technology, Tongji University, Shanghai, 200092 China; 2 School of Pharmacy, Hangzhou Normal University, Hangzhou, 311121 Zhejiang China; 3 Shuangming Biotechnology (Suzhou) Co., Ltd., Suzhou, 215000 Jiangsu China

**Keywords:** Peptide, Protein identification, Nanopore sensing, Post-translational modification

## Abstract

Effective and precise detection of peptides and their post-translational modifications (PTMs) is crucial for biomedical research but remains technically challenging. Here, we present a nanopore sensing strategy that enables label-free analysis of negatively charged peptides. By introducing arginine residues into the nanopore and using amino-cyclodextrin as a molecular adaptor, the system enables unambiguous discrimination of short negatively charged homopeptides differing by only a single residue. Building on this capability, the platform further demonstrates high sensitivity for detecting representative PTMs, including acetylation, lactylation, and phosphorylation, introduced on a negatively charged peptide scaffold. Notably, the system could also analyze fragments generated by proteolytic digestion of full-length proteins, underscoring its potential for accurate protein identification. This work offers an accessible and reliable strategy to characterize peptide variants with single-amino acid differences and PTMs, which may facilitate future applications in peptide biomarker detection and protein sequence analysis.

**Graphical abstract:**

**Figure:**
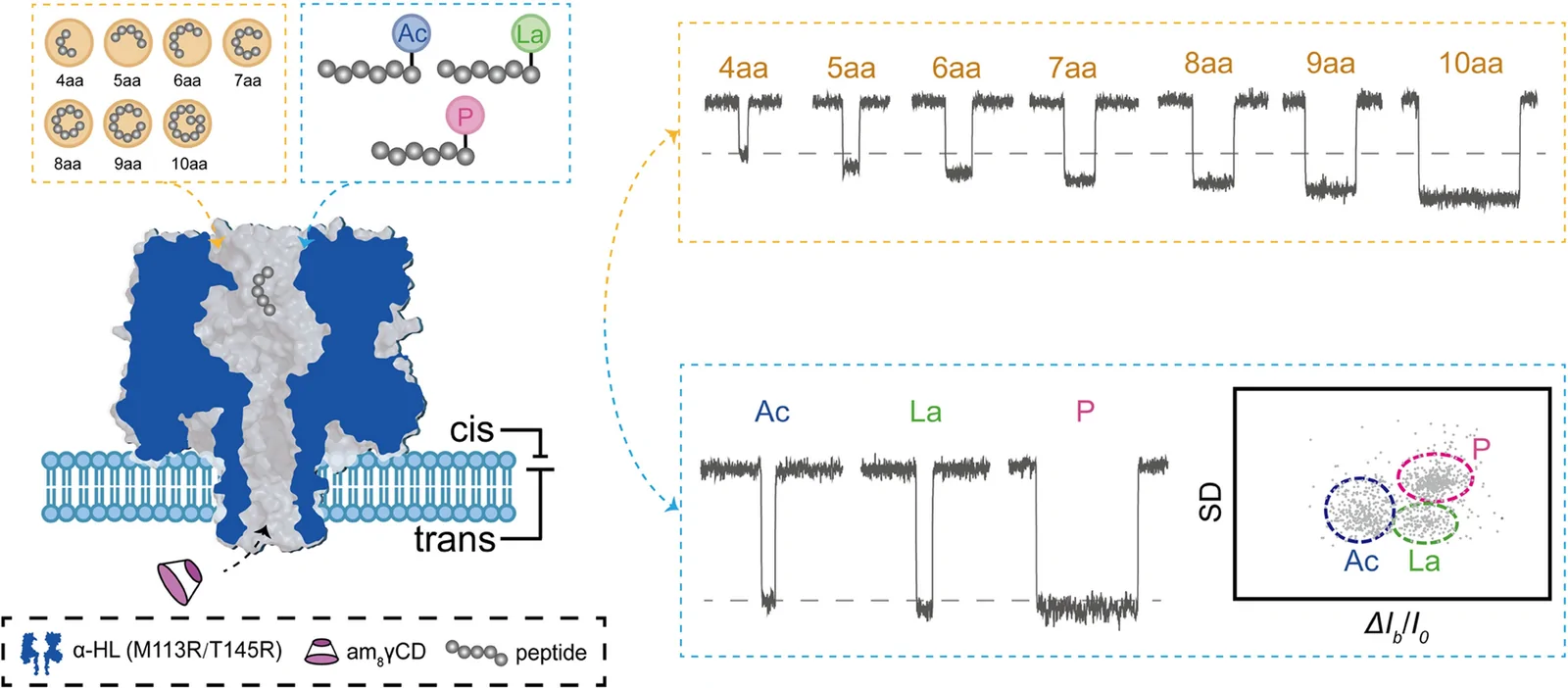

**Supplementary Information:**

The online version contains supplementary material available at https://doi.org/10.1186/s12951-026-04818-z.

## Introduction

Peptides play essential roles in various biological processes, including cellular signaling, immune responses, and metabolic regulation [1, 2]. Subtle alterations in amino acid sequences or aberrant post-translational modifications (PTMs) can disrupt their biological functions and contribute to disease. For example, sequence variations in amyloid-β peptides have been linked to familial Alzheimer’s disease [3, 4], while abnormal PTMs, such as phosphorylation, acetylation, and lactylation, have been implicated in diverse pathological processes, including neurodegeneration, tumor progression, and inflammation [5]. Currently, peptide analysis mainly relies on mass spectrometry (MS) [6, 7] and immunoassay-based strategies [8]. While these techniques have contributed significantly to the field of peptidomics, they still face important analytical limitations in sensitivity and reproducibility. MS-based methodologies remain limited in their detection sensitivity for low-abundance peptides and suffer from inherent variability in ionization efficiency across different peptides [9, 10]. Immunoassays, which rely on antibodies to selectively detect peptides, are constrained by the challenges of antibody development, extensive sample preparation, and poor reproducibility [11]. These challenges motivated continued development of complementary analytical strategies for peptide characterization.

Nanopore technology, mainly known as a promising single-molecule platform for DNA and RNA sequencing, has been attracting increasing attentions for the study of peptides and proteins [12–16]. By monitoring characteristic ionic current changes generated when individual molecules translocate through, reside within, or interact transiently with a nanopore, this technique enables direct, label-free characterization of target molecules. Based on this, nanopore sensing has been applied to characterize essential features of proteins and peptides, such as size [17], mass [18], charge [19, 20], post-translational modifications [21–29], and subtle chemical/structural differences [30–32]. It has also been used to effectively monitor dynamic processes of proteins and peptides [33], including unfolding kinetics [34, 35], conformational changes [36, 37], protein-protein or protein-ligand interactions [38, 39], and enzymatic reactions [40]. In recent years, controlled ratcheting motion of target peptides and proteins has been achieved with the assistance of DNA-processive motors [41–43] or protein unfoldases [44]. Moreover, several proof-of-concept studies have demonstrated that nanopore sensors can identify individual amino acids [45], suggesting their potential for peptide sequence analysis [46–48]. Beyond these advances in fundamental peptide and protein analysis, nanopore sensing has also shown promise in biomarker detection [13, 24], highlighting potential clinical and point-of-care diagnosis.

Here, we focus on the analysis of short peptides, as discriminating subtle compositional differences in short peptides is a fundamental analytical challenge for biomarker discovery and protein sequencing. Previous nanopore studies have reported promising results in discriminating peptides with single-amino-acid-level differences [17, 18, 32, 49–51], including a recent demonstration of peptide biomarker discrimination in serum [52]. Considering that negatively charged peptides constitute a biologically meaningful subset of the peptidome, we explore the capability of nanopore sensors to distinguish short negatively charged peptides with single-amino acid resolution. In this study, we employed a host-guest interaction-assisted nanopore sensing strategy, in which cyclodextrin is used as a molecular adaptor. Cyclodextrins are widely used in nanopore sensing since they can be noncovalently lodged within the nanopore lumen, thereby creating a confined recognition site for guest analytes [53–56]. The reversible interactions between cyclodextrin and target molecules generate characteristic ionic current signals, enabling the detection and discrimination of various analytes [57, 58]. Here, negatively charged homopeptides with different lengths were unambiguously identified and distinguished by introducing polyamine-decorated cyclodextrin as the recognition adaptor in a mutant α-hemolysin nanopore (M113R/T145R). This sensing strategy also exhibited high sensitivity to post-translational modifications on peptides, such as acetylation, lactylation, and phosphorylation. Furthermore, we applied this method to analyze the digestion products of three model proteins, demonstrating its potential for accurate protein identification at the single-molecule level.

## Materials and methods

### Materials

Peptides (Table S1) were custom-synthesized by DG peptides Co., Ltd. The S100B, S100A1, and S100A9 proteins were expressed and purified in our laboratory. *E. coli* BL21 (DE3) was purchased from Beijing Qingke Biotech Co., Ltd. Yeast extract and tryptone for medium preparation were obtained from OXOID. Sodium chloride (NaCl), potassium chloride (KCl), and imidazole were purchased from Sinopharm Chemical Reagent Co., Ltd. (China). Octakis-(6-amino-6-deoxy)-γ-cyclodextrin (am_8_γCD) was purchased from GlpBio. Isopropyl β-D-1-thiogalactopyranoside (IPTG) was sourced from BBI Life Sciences. Kanamycin and 4-(2-hydroxyethyl)-1-piperazineethanesulfonic acid (HEPES) were acquired from Shanghai YuanYe Biotechnology. 1,2-Diphytanoyl-sn-glycero-3-phosphocholine (DPhPC) was obtained from Avanti Polar Lipids. Hexadecane and pentane were sourced from Sigma-Aldrich. DL-Dithiothreitol (DTT) was purchased from Solarbio. Guanidine hydrochloride (GdnHCl) was acquired from Sangon Biotech (Shanghai) Co., Ltd. The trypsin was purchased from Cienry. Ultrafiltration tubes (5 kDa, 10 kDa) were purchased from Biosharp.

### Buffer preparation

The buffer solution used for nanopore measurements is 1 M KCl (1 M KCl, 10 mM Tris at pH 7.5). The buffer was prepared using Milli-Q water and filtered through a 0.2 μm Whatman membrane prior to use.

### α-HL expression and purification

For expression of α-HL protein, the genes coding for WT and M113R/T145R with a C-terminal 6 × His tag were respectively cloned into the pET-30a( +) plasmid (GenScript). The WT and M113R/T145R plasmids were transformed into *E. coli* BL21 (DE3) cells. Successfully transformed *E. coli* cells were grown in LB medium until an OD600 of 0.6-0.8 was reached. Then, protein expression was induced by adding IPTG to a final concentration of 1 mM and incubating at 16 °C and 200 rpm overnight. The cells were then harvested, resuspended in lysis buffer (500 mM NaCl, 20 mM HEPES, 0.1% (w/v) Triton X-100, pH 8.0), and lysed using a sonicator for 10 min. The supernatant was collected and applied to a nickel affinity column. Nonspecifically bound proteins were first removed by washing the column with a buffer containing 0.5 M NaCl, 20 mM HEPES, 5 mM imidazole (pH 8.0). Subsequently, the target α-HL protein was eluted using a linear gradient of imidazole into the elution buffer (0.5 M NaCl, 20 mM HEPES, 500 mM imidazole, pH 8.0).

### Preparation of S100B, S100A1, and S100A9 proteins

The reference sequences for the S100B, S100A1, and S100A9 proteins were obtained from Uniprot (protein accession numbers P04271, P23297, and P06702, respectively). The genes encoding S100B, S100A1, S100A9 with a C-terminal 6 × His tag were cloned into the pET-30a( +) plasmid. Then the target proteins were expressed in *E. coli* BL21 (DE3) and purified by affinity chromatography, following the detailed experimental procedures for α-HL nanopore preparation described in Methods.

### Protein digestion

0.5 mg of S100B, S100A1, and S100A9 were initially denatured in the presence of 3.6 M guanidine hydrochloride (GdnHCl) and 20 mM DTT at 37 °C for 1 h with continuous stirring at 200 rpm. Here, DTT was added to prevent disulfide bond formation from interfering with protein digestion. After the denaturation, the proteins were buffer-exchanged into PBS using an ultrafiltration tube with 2 washing steps. Subsequently, each protein was digested overnight with trypsin at a 1:20 mass ratio (protein:trypsin) at 37 °C. The digestion products were filtered using a 10 kDa ultrafiltration tube to remove the residual trypsin and were analyzed with nanopores within the same day.

### Nanopore measurements and data analysis

All nanopore measurements were performed as previously reported [59]. Typically, the measurement setup consists of two polyoxymethylene chambers separated by a Teflon membrane containing an aperture of approximately 100 μm in diameter. Prior to each measurement, the aperture was pretreated with a mixture of hexadecane and pentane (v/v = 1:200). Each chamber was then filled with 500 μL of electrolyte solution (1 M KCl, 10 mM Tris, pH 7.5) using a micropipette. Two Ag/AgCl electrodes were immersed in each chamber and interfaced with an Axon 200B patch-clamp amplifier (Molecular Devices) to form a closed circuit. We defined the grounded chamber as *cis* compartment and the voltage-applied chamber as *trans* compartment. After adding a drop of pentane solution of DPhPC (5 mg/mL) into each chamber, the lipid bilayer was formed by pipetting the electrolyte buffer up and down several times. Subsequently, the α-HL (M113R/T145R) nanopores were introduced into the *cis* side. Following the insertion of a single α-HL (M113R/T145R) nanopore, the *cis* chamber solution was immediately replaced with fresh buffer to prevent further pore insertions. All single-channel measurements were carried out using an Axon 200B patch-clamp amplifier and digitized by a Digidata 1550B digitizer (Molecular Devices).

Prior to peptide measurements, am_8_γCD was added to the *trans* compartment at a final concentration of 40 μM. For nanopore sensing of peptides under positive bias, the peptides were added to the *cis* chamber unless stated otherwise. K_10_, which is positively charged, was instead added to the *trans* chamber. For W10, the peptide was first dissolved in 5% DMSO and then added to the cis side to a final concentration of 50 μM, at which no visible precipitation was observed. For the detection of proteolytically cleaved peptides, protein digestion products were prepared according to Methods, and then were added to the *cis* compartment at a final concentration of 20 ng/μL. All nanopore measurements were carried out in the buffer of 1 M KCl, 10 mM Tris at pH 7.5. A constant potential of + 60 mV was continually applied during measurements, and current traces were continually recorded with a 25 kHz sampling rate and low-pass filtered at 5 kHz.

All nanopore events were detected by Clampfit 10.7, and characteristic parameters of each event including *ΔI*_*b*_*/I*_*0*_ and SD were extracted. To minimize interferences caused by noise events, events with dwell time < 2 ms were ignored. The events acquired from peptides KD_4_S, (KD_4_S)-Ac, (KD_4_S)-La and (KD_4_S)-P were subjected to cluster analysis using DBSCAN to remove noise events. For nanopore results acquired from digestion products (Fig. 4), the data processing and clustering analysis were performed using a custom Python program. Before clustering, outlier removal was performed in the two-dimensional feature space defined by the standard deviation (SD) and *ΔI*_*b*_*/I*_*0*_ using the Isolation Forest algorithm (with the contamination parameter set to 0.10), and the identified outlier events were excluded from the subsequent clustering analysis. The clustering analysis was then performed using a two-stage K-means strategy. In the two-dimensional feature space, events were first partitioned into high- and low- regions along the feature *ΔI*_*b*_*/I*_*0*_. Events in the high-density region were then subjected to one-dimensional K-means clustering along this feature to resolve distinct sub-populations within the main peak, whereas events in the low-density region were clustered in the standardized two-dimensional space of *ΔI*_*b*_*/I*_*0*_ and SD, with the optimal number of clusters determined by maximizing the silhouette coefficient. To quantify the spatial extent and relative crowding of each cluster, we computed the mean vector μᵢ and covariance matrix Σᵢ of the event coordinates in the two-dimensional feature plane and constructed a Gaussian confidence ellipse for each cluster. The semi-axes of each ellipse were determined from the eigenvalues of Σᵢ and the χ^2^ critical value with 2 degrees of freedom, and the ellipse area was denoted Aᵢ. On this basis, we defined a density index:

$${\uprho}_{i}=\left({n}_{i}/N\right)/{A}_{i}$$

where nᵢ is the number of events in the i-th cluster and N is the total number of events in the dataset. The custom machine learning code, which is referred to as Protein Digestion Product Classifier, is shared on figshare. Follow the link https://figshare.com/s/5b1cda502051c5fc7c53 to download.

## Results and discussion

### Discrimination of glutamate homopeptides at single-amino acid resolution

**Fig. 1:**
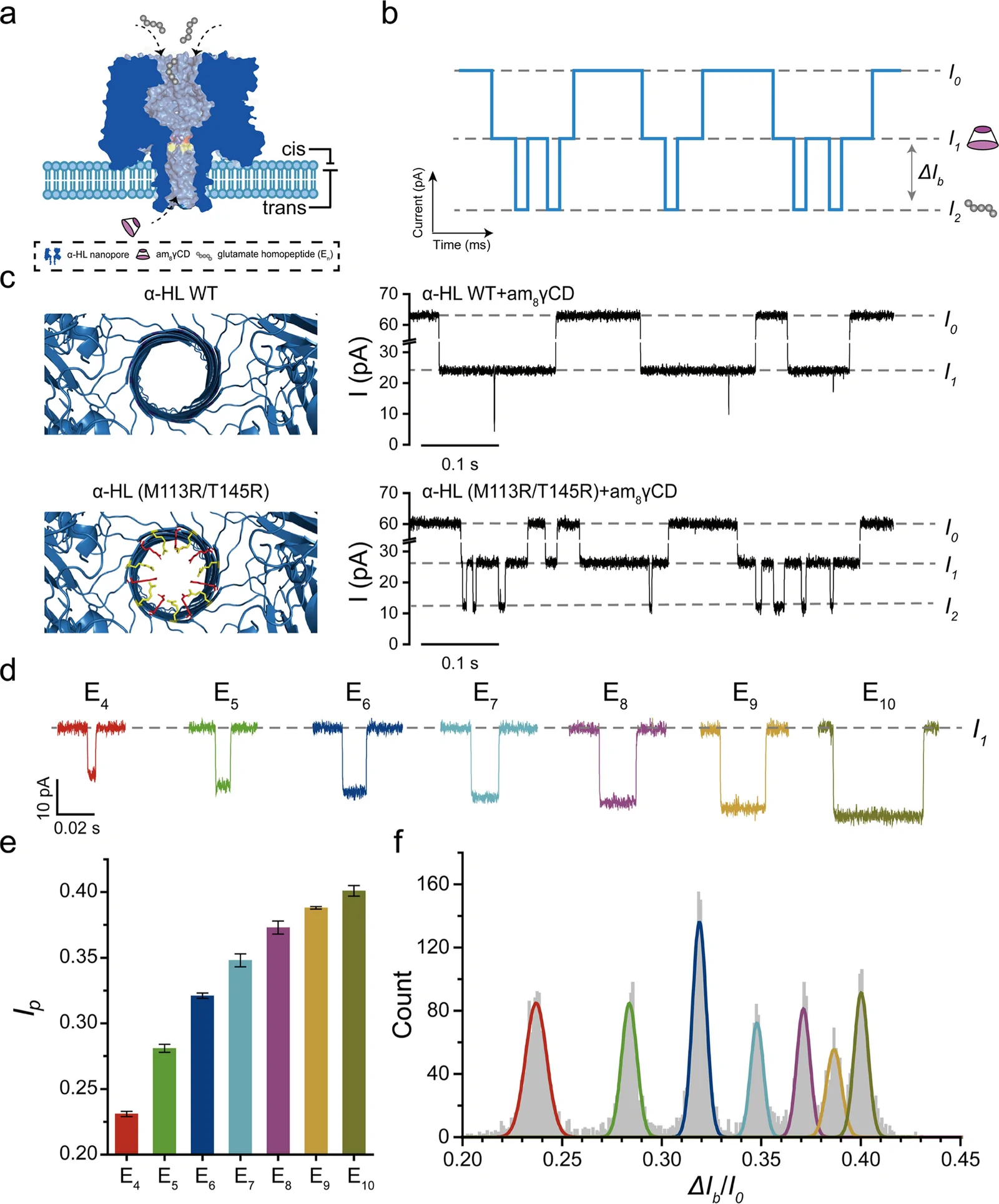
Discrimination of glutamate homopeptides (E_n_) at single-amino acid resolution with am_8_γCD as the adaptor. (**a**) Schematic illustration of peptide detection using nanopore measurements. The am_8_γCD is employed as a molecular adaptor for peptides. Upon noncovalent attachment of am_8_γCD within the α-HL nanopore, the translocation of glutamate homopeptides generates detectable current signals. (**b**) Schematic representation of the expected nanopore current trace during peptide detection. *I*_*0*_, current level of the unoccupied α-HL nanopore; *I*_*1*_, current level corresponds to am_8_γCD bound within the nanopore; *I*_*2*_, current level corresponds to the peptide binding to the nanopore-am_8_γCD complex. (**c**) Top-down views of the nanopore lumen of α-HL WT and α-HL (M113R/T145R) (left), and representative current traces of glutamate homopeptide E_4_ translocating through the α-HL WT and α-HL (M113R/T145R) in the presence of am_8_γCD (right). (**d**) Representative nanopore events of glutamate homopeptides of seven different lengths (4,5,6,7,8,9, and 10 amino acids). The current level *I*_*1*_ is marked with a dashed line. The events from each peptide (E_4_ to E_10_) are shown in different colors. (**e**) Mean and standard deviations of *I*_*p*_ (mean blockade current, Figure S13) acquired from three independent measurements for each peptide. (**f**) Histogram of *ΔI*_*b*_*/I*_*0*_ values corresponding to the current trace for simultaneous detection of all seven glutamate homopeptides (*N* = 5760). The histogram demonstrates seven well-resolved peaks, each corresponding to an individual glutamate homopeptide. All peaks were fitted with Gaussian functions. Different peptides were simultaneously added to the *cis* side, each at a final concentration of 0.4 μM. All measurements were carried out in the buffer of 1 M KCl, 10 mM Tris at pH 7.5

To construct the peptide sensing system, octakis-(6-amino-6-deoxy)-γ-cyclodextrin (am_8_γCD) was introduced into an α-HL nanopore as the recognition adaptor (Fig. 1a). Once lodged within the nanopore, am_8_γCD provided a confined host site for reversible peptide interactions. It is expected that the host-guest inclusion of am_8_γCD with peptides could generate detectable ionic current signatures and permit continuous measurement of different peptides. Binding of am_8_γCD to the α-HL nanopore from the *trans* compartment would give rise to a stable current blockade (*I*_*1*_) (Fig. 1b). Upon subsequent addition of peptides to the *cis* compartment, a secondary blockade level (*I*_*2*_) could then arise from the current level of am_8_γCD. To characterize the peptide-induced blockade events, the relative current blockade could be defined as *ΔI*_*b*_*/I*_*0*_, where *ΔI*_*b*_ = *I*_*2*_*-I*_*1*_.

To evaluate the am_8_γCD-assisted nanopore sensing strategy for peptides, a set of glutamate homopeptides (denoted as E_n_, where ‘n’ represents the number of glutamate residues) were designed and synthesized (Table S1). The wild-type α-HL nanopore (α-HL WT, Figure S1-S2) was first employed to analyze peptides in the presence of am_8_γCD, but only transient spiky events were observed upon addition of peptide E_4_ (Fig. 1c, Figure S3). These short-lived events suggest that the noncovalent peptide-pore interactions (e.g., electrostatic and hydrogen-bonding interactions) are insufficient to stabilize long-lived residence of the peptide within the pore under the present experimental conditions. To address this, two arginine mutations were introduced at the lumen-facing positions M113 and T145 (Figure S4-S6), where M113 is a well-established engineering site near the constriction in α-HL [60, 61], and T145 provides a complementary position for tuning the local electrostatic and steric environment. The positively charged arginine residues increase the pore’s anion selectivity and are therefore expected to strengthen the electroosmotic flow (EOF), which is directed from *cis* to *trans* under positive bias. For negatively charged homopeptides, both the electrophoretic force (EPF) and EOF act in the same direction under positive bias (Figure S7), thereby facilitating peptide capture and entry into the nanopore. As expected, resolvable characteristic blockade events were observed for E_4_ in the α-HL (M113R/T145R)-am8γCD sensor (Fig. 1c, Figure S8). The measurements were performed in 1 M KCl buffer (1 M KCl, 10 mM Tris at pH 7.5). With increasing applied voltage, the events exhibited shorter dwell times (Figure S9), indicating that the peptides translocate through the nanopore. To further investigate the interactions between the peptide and the nanopore-am8γCD sensor, molecular docking analysis was performed (Figure S10). Peptide E_4_ showed a favorable affinity score of −7.0 with the nanopore-am8γCD sensor, accompanied by the formation of multiple hydrogen bonds and salt bridges (Table S2). Besides, molecular docking analysis was also performed using the α-HL (M113R/T145R)-γCD sensor (Figure S10). In this case, E_4_ showed a weaker affinity score of −6.5, with fewer hydrogen bonds and salt bridges. These results suggest that amino modification of γCD may strengthen the peptide-sensor interactions, thereby contributing to the improved peptide-sensing performance of the nanopore-am8γCD sensor.

To evaluate the peptide-discriminating ability of the engineered α-HL nanopore (M113R/T145R, hereafter termed α-HL) equipped with am_8_γCD, we tested seven glutamate homopeptides (peptides E_4_ to E_10_, Table S1, Figure S11), each differing by a single glutamate residue in length (4,5,6,7,8,9, and 10 amino acids). As demonstrated by their corresponding nanopore events (Fig. 1d), the blockade signals of each peptide are fully distinguishable by the current amplitude. Generally, the blockade current amplitude exhibits a positive correlation with the peptide length. Among them, peptide E_10_ produced the deepest blockade signal, while peptide E_4_ exhibited the shallowest. The dwell-time distributions of E_4_-E_10_ were further analyzed and are shown in Figure S12. To evaluate the reproducibility of this method, three independent measurements were performed for each peptide, and the corresponding mean blockade current (*I*_*p*_, as defined in Figure S13) was calculated and summarized (Fig. 1e, Table S3). Furthermore, a simultaneous measurement of all seven glutamate homopeptides was performed, resulting in seven types of blockade currents corresponding to the nanopore detection of peptides E_4_ to E_10_ (Figure S14). The event histogram of *ΔI*_*b*_*/I*_*0*_ also demonstrated seven well-resolved peaks (Fig. 1f), highlighting the capability of the am_8_γCD-assisted α-HL nanopore to discriminate glutamate homopeptides with single-amino acid resolution. Impressively, the nanopore sensor equipped with am_8_γCD even exhibited sufficient sensitivity to detect peptides composed of as few as two or three glutamate residues (E_2_ and E_3_, Figure S15).

Previous nanopore studies have reported different strategies for peptide analysis. Maglia and co-workers developed a FraC-based peptide mass readout by using pore variants with different diameters to accommodate peptides of different sizes [18]. In comparison, our am_8_γCD-assisted nanopore sensing strategy discriminates glutamate homopeptides of different lengths using a single engineered α-HL nanopore under the same experimental conditions (Table S4). Notably, even the dipeptide E_2_ produced measurable events with a dwell time of 3.72 ms (Figure S15). Zhang et al. reported a cucurbit[7]uril-mediated host-guest strategy that distinguishes the 20 proteinogenic amino acid residues after they are incorporated into designed phenylalanine-containing peptide probes [48]. In this approach, the variable residue is placed at a particular position within the probe peptide to generate distinguishable nanopore signals, whereas our method can discriminate target peptides without requiring their sequence differences to be located at a fixed position or involving any labeling.

### Length-dependent identification of aspartate homopeptides

To further assess the capability of the nanopore-am_8_γCD sensor in identifying peptides of different lengths, a series of aspartate homopeptides were prepared and analyzed. Specifically, the homopeptides consisted of 4 to 10 aspartate residues and were designated as peptides D_4_ to D_10_ (Figure S16, Table S1), respectively. Aspartate residues, bearing a negatively charged carboxyl side chain, were also expected to interact strongly with the (M113R/T145R) -am_8_γCD sensor, similar to glutamate residues.

**Fig. 2:**
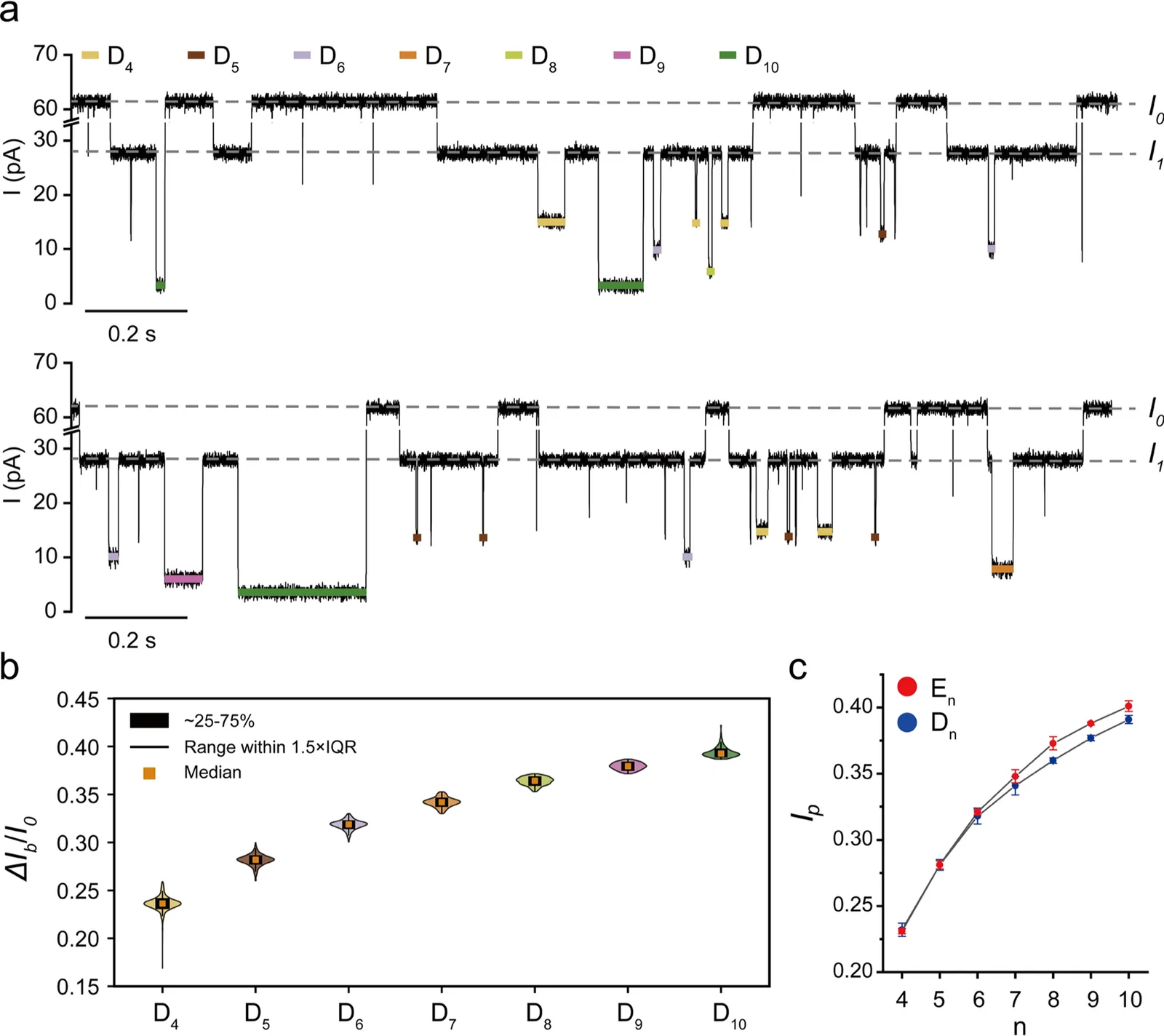
Discrimination of aspartate homopeptides (D_n_) differing by a single amino acid in length. (**a**) Representative current traces acquired during simultaneous sensing of all seven aspartate homopeptides (D_4_ to D_10_). Events from different peptides can be clearly identified from the trace according to the characteristic blockade depth and were marked with corresponding color bars. Aspartate homopeptides were simultaneously added to the *cis* compartment, each at a final concentration of 0.4 μM. A constant voltage of +60 mV was continually applied during the measurement. (**b**) The violin plot of *ΔI*_*b*_*/I*_*0*_ of events (*N* = 6316) acquired as described in a. Seven distinct distributions were observed, each colored violin corresponding to one aspartate homopeptide. IQR, interquartile range. (**c**) Comparison of *I*_*p*_ (mean blockade current) of E_n_ and D_n_. The two homopeptides E_n_ and D_n_ showed nearly identical *I*_*p*_ values at n = 4-6, while a gradual divergence appeared starting from n = 7. As n increases, E_n_ exhibits slightly higher *I*_p_ values than D_n_

During the simultaneous measurement of peptides D_4_ to D_10_, seven distinct types of blockade events were recorded (Fig. 2a). These events are more clearly illustrated in Figure S17, which presents representative examples of each individual event type. The distribution of *ΔI*_*b*_*/I*_*0*_ for peptides D_4_ to D_10_ is shown in a violin plot (Fig. 2b), revealing seven distinct distributions that correspond to the different blockade event types. To confirm that each observed population originated from a specific aspartate homopeptide, three independent measurements were performed for each peptide. The mean blockade current values (*I*_*p*_ ± SD) for peptides D_4_ to D_10_ were determined as follows (Table S3, Figure S18): 0.232 ± 0.005, 0.281 ± 0.004, 0.318 ± 0.006, 0.341 ± 0.007, 0.361 ± 0.002, 0.377 ± 0.002, and 0.390 ± 0.003. These mean blockade currents matched those of each peptide population in the mixture measurements, confirming that D_4_ to D_10_ correspond to distinct event groups. The blockade current amplitude showed a clear dependence on peptide length, with D_4_ producing the shallowest blockades and D_10_ the deepest. In addition, the dwell-time distributions of D_4_-D_10_ were further analyzed and are shown in Figure S19. These results demonstrate the capability of the nanopore-am_8_γCD sensor in length-based discrimination of aspartate homopeptides with single-amino acid resolution. Furthermore, we compared the mean blockade currents of glutamate (E) and aspartate (D) homopeptides. As shown in Fig. 2c, the *I*_*p*_ values of the E_n_ peptides are systematically higher than those for their D_n_ counterparts when n ≥ 7. This difference can be attributed to the larger side-chain volume of glutamate residue (E), which results in a greater degree of current blockade.

Beyond negatively charged peptides, we also investigated positively charged and neutral homopeptides. Three representative homopeptides, each composed of ten lysine, glycine, or tryptophan residues, respectively (termed K_10_, G_10_ and W_10_, Figure S20), were prepared and characterized by nanopore. All three peptides generated measurable blockade events. However, their current signals displayed broad amplitude distributions (Figure S21), in contrast to the uniform blockade levels recorded for aspartate homopeptides. These results suggest that the nanopore-am_8_γCD sensor can detect positively charged and neutral peptides. However, the achievement of single-amino acid resolution, as observed for aspartate and glutamate homopeptides, may depend more strongly on the balance between EPF and EOF during peptide transport (see the expected force directions in Figure S7).

Based on the peptide-sensing capability of this strategy, we further explored the potential application of the nanopore-am8γCD sensor in synthetic peptide quality control. D_8_ was selected as a model peptide and thermally treated to induce degradation. Nanopore analysis of the treated sample showed five major *ΔI*_*b*_*/I*_*0*_ populations (Figure S22), corresponding to D_4_, D_5_, D_6_, D_7_, and intact D_8_, respectively. This assignment was supported by mass spectrometry analysis (Figure S23), which confirmed the presence of D_4_-D_8_ species in the same sample. In addition to the D_8_ model peptide, bivalirudin, a synthetic anticoagulant peptide drug with the sequence FPRPGGGGNGDFEEIPEEYL, was also tested. Intact bivalirudin mainly produced events from the open-pore current level (Figure S24), with only a few further blockades from the am8γCD-associated current level, likely due to limited size compatibility with the cyclodextrin adaptor. After thermal treatment, the am8γCD-associated events increased (Figure S24), suggesting that shorter peptide fragments generated by thermal degradation were detected by the nanopore-am8γCD sensor. Mass spectrometry further confirmed the degradation of bivalirudin after thermal treatment (Figure S25). These results suggest that the am8γCD-assisted nanopore sensing strategy can contribute to synthetic peptide quality control by evaluating peptide degradation.

### Identification of PTMs using the nanopore-am_8_γCD sensor

**Fig. 3:**
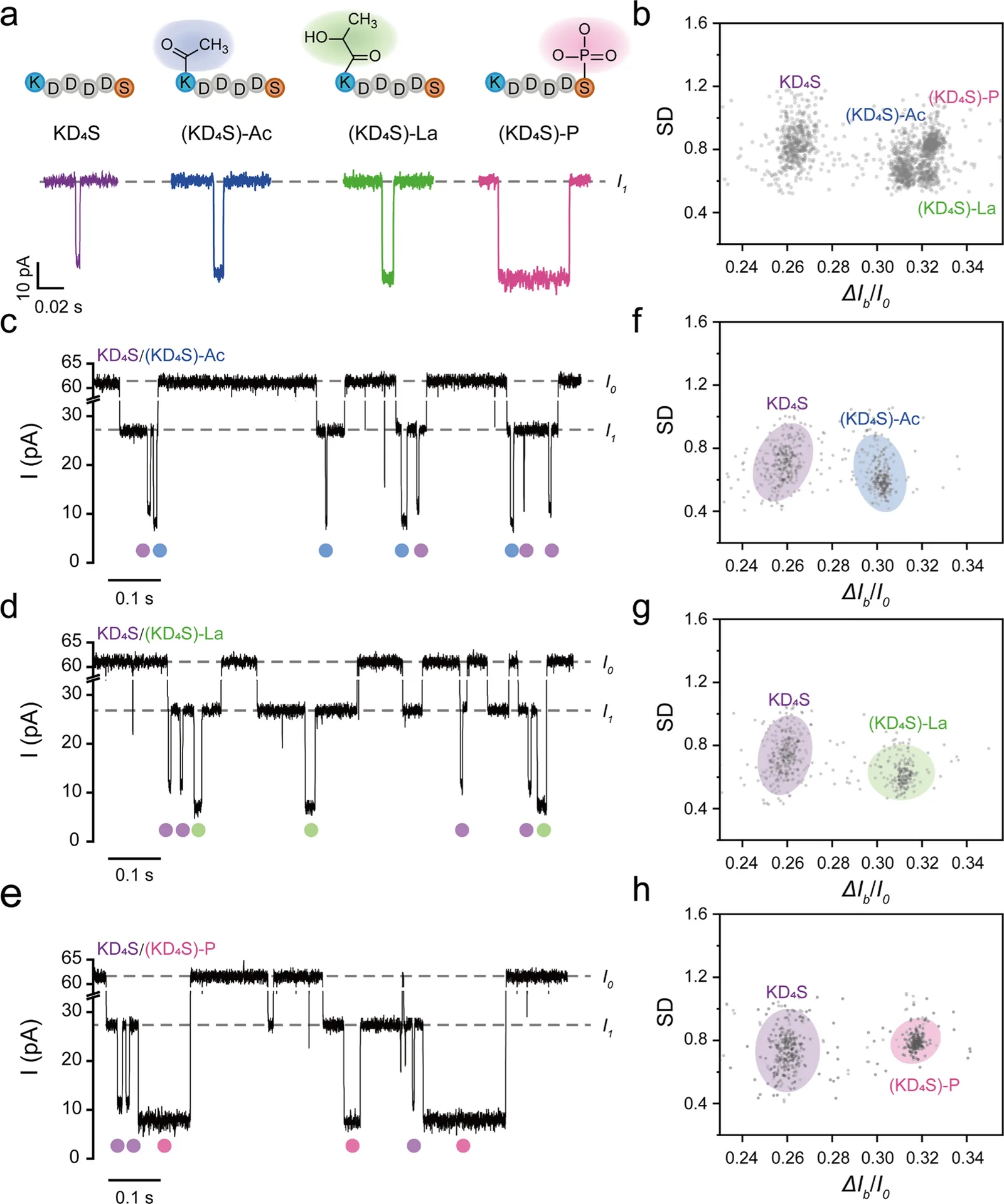
Identification of peptides with PTMs. (**a**) Top: composition of KD_4_S and peptides with PTMs, including acetylation ((KD_4_S)-Ac), lactylation ((KD_4_S)-La) and phosphorylation ((KD_4_S)-P). Bottom: representative nanopore events of corresponding peptides. (**b**) The scatter plot of standard deviation versus *ΔI*_*b*_*/I*_*0*_ of events (*N* = 1460) produced by the four peptides described in a. During the measurement, different peptides were simultaneously added to *cis* chamber at a final concentration of 0.4 μM for each peptide. (**c-e**) Representative current traces acquired from simultaneous sensing of KD_4_S/(KD_4_S)-Ac (**c**), KD_4_S/(KD_4_S)-La (**d**) and KD_4_S/(KD_4_S)-P (**e**). Each peptide was added to the *cis* compartment at a final concentration of 0.4 μM. Events were identified and marked with purple (KD_4_S), blue ((KD_4_S)-Ac), green ((KD_4_S)-La) and pink symbols ((KD_4_S)-P). (**f-h**) Scatter plots of standard deviation versus *ΔI*_*b*_*/I*_*0*_ of events produced by KD_4_S/(KD_4_S)-Ac (**f**, *N* = 636), KD_4_S/(KD_4_S)-La (**g**, *N* = 556) and KD_4_S/(KD_4_S)-P (**h**, *N* = 890). In each plot, the events produced by KD_4_S and modified peptides were fully distinguished. The data in the plot were processed using DBSCAN (epsilon = 0.04, min_samples = 10). The colored ellipses represent 85% confidence regions for the corresponding event populations

PTMs are covalent chemical modifications that occur on proteins after translation and can alter the physicochemical properties of peptides [62]. Given that our nanopore-am_8_γCD sensor generated characteristic current signatures for negatively charged peptide backbones, we used a negatively charged peptide scaffold as a template onto which PTMs were introduced in order to evaluate the ability of the platform to detect and distinguish these modifications. Specifically, we designed three model PTM-containing peptides derived from the sequence KDDDDS (referred to as KD_4_S), featuring acetylation or lactylation at the lysine residue, or phosphorylation at the serine residue. These peptides were designated as (KD_4_S)-Ac, (KD_4_S)-La, and (KD_4_S)-P (Fig. 3a, Figure S26), respectively.

When measured using the nanopore-am_8_γCD sensor, the modified peptides produced characteristic current events that were clearly distinguishable from those of the unmodified peptide KD_4_S (Fig. 3a). Event scatter plots of standard deviation versus *ΔI*_*b*_*/I*_*0*_ from three independent measurements for each peptide were generated (Figure S27), demonstrating the consistency of the measurements. To minimize background noise events, the scatter plot data were processed using Density-Based Spatial Clustering of Applications with Noise (DBSCAN), a density-based clustering algorithm that identifies and removes low-density outliers. When all four peptides were analyzed using the same pore, the resulting scatter plot exhibited four distinct populations (Fig. 3b), each corresponding to one of the peptides. Furthermore, simultaneous detection of KD_4_S and each modified peptide was carried out individually in separate measurements (Fig. 3c-e). In each case, two clearly separated signal populations were observed (Fig. 3f-h), confirming the capability of the nanopore-am_8_γCD sensor to resolve different peptide modifications.

To further assess the quantitative performance of the nanopore-am8γCD sensor, mixed samples containing (KD_4_S)-P and (KD_4_S) were analyzed. Since the two peptides exhibited different capture efficiencies, a calibration curve was established to correlate the observed event-count ratios with the actual molar ratios (Figure S28). Based on this calibration, the (KD_4_S)-P/(KD_4_S) molar ratios obtained from four independently prepared mixed samples showed good agreement with the actual values. These results demonstrate that the nanopore-am8γCD sensor can effectively distinguish peptide modifications and further provide a practical calibration-based strategy for quantitative analysis of modified and unmodified peptide mixtures.

### Protein profiling through nanopore sensing of proteolytically cleaved peptides

**Fig. 4:**
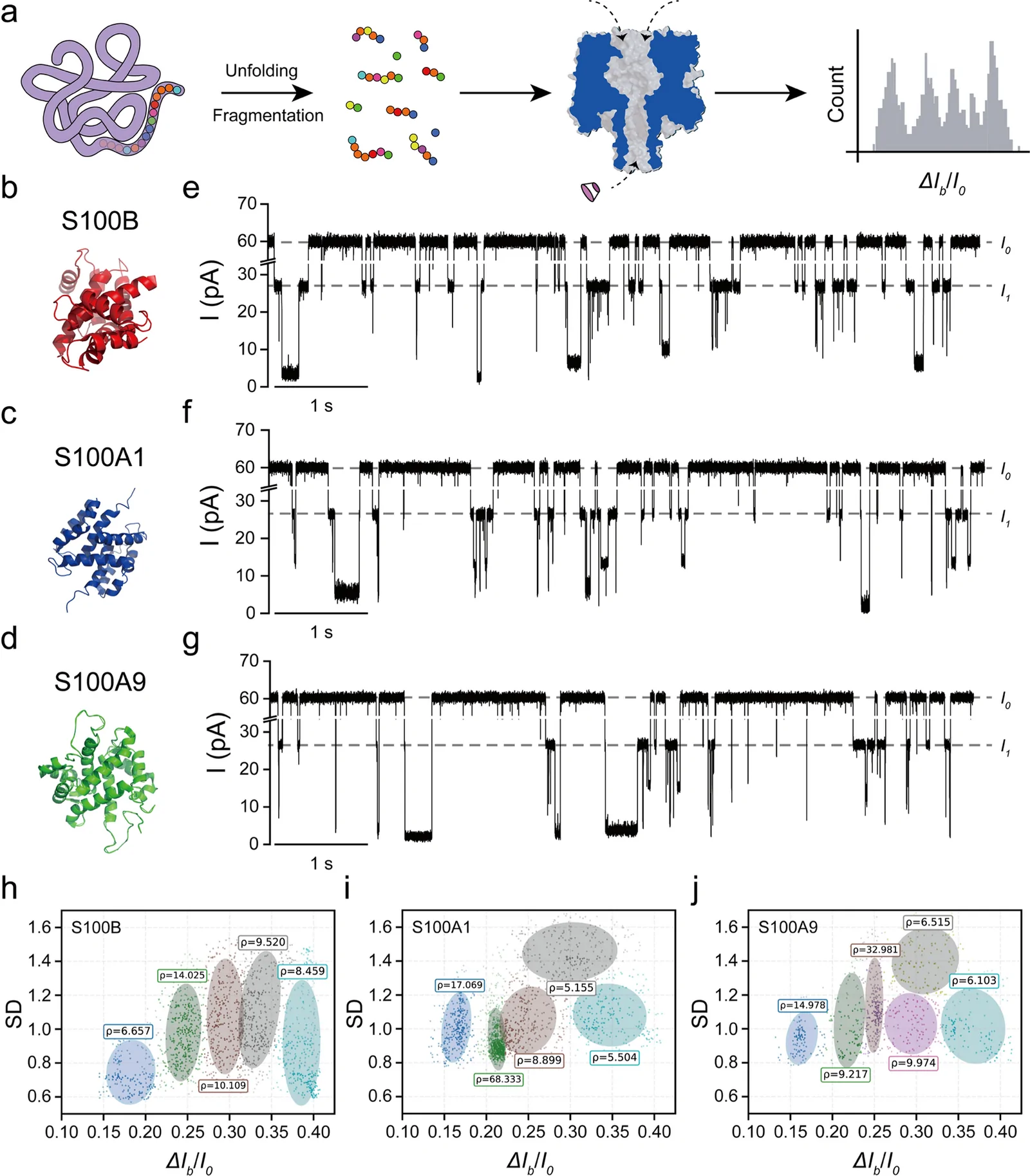
Detection of proteolytically cleaved peptides by nanopore. (**a**) The schematic diagram of the detection of proteolytically cleaved peptides from protein using nanopore. The target protein was initially digested by trypsin into peptide fragments. The generated peptides were subsequently analyzed with an α-HL nanopore-am_8_γCD sensor, enabling identification of proteins. Schematic diagram of the structures of the S100B (**b**), S100A1 (**c**) and S100A9 (**d**). Representative current traces of nanopore measurement of the digestion product from S100B (**e**), S100A1 (**f**) and S100A9 (**g**). Scatter plots of the standard deviation versus *ΔI*_*b*_*/I*_*0*_ for nanopore events corresponding to proteolytic peptide fragments of S100B (**h**, *N* = 2418), S100A1 (**I**, *N* = 1928), and S100A9 (**j**, *N* = 1562). Noticeable differences in the distribution patterns were observed, enabling clear identification of different proteins based on their corresponding distributions. Distinct event populations were identified using the k-means clustering algorithm, with 1σ covariance error ellipses shown to indicate the core distribution region of each cluster. Theoretically, each ellipse covers approximately 68% of the data points. The label ρ denotes the relative density of each cluster, defined as the fraction of events assigned to that cluster normalized by the area of the corresponding ellipse

Accurate detection of proteins is essential for understanding biological functions, elucidating disease mechanisms and promoting therapeutic development. Given the fact that developing a universal strategy for protein characterization is challenging due to their complexity, we assessed the feasibility of our nanopore-am_8_γCD sensor to detect cleaved peptide fragments as an alternative approach for protein detection. Trypsin, a highly specific protease that cleaves peptide bonds after lysine or arginine residues, was used to digest different target proteins into peptide fragments. Subsequent nanopore measurements of these digestion products produced distinct characteristic signals, enabling identification of various proteins (Fig. 4a).

Among the proteins examined, we selected members of the S100 family as representative model proteins. The S100 family comprises a group of low-molecular-weight calcium-binding proteins that plays essential roles in the regulation of various cellular processes[63]. Importantly, these proteins display pronounced negatively charged character, and their proteolytic cleavage generates peptide fragments enriched in negatively charged residues. Therefore, S100 proteins provide a suitable native system for extending our negative charged peptide detection strategy to protein-derived samples. Accordingly, three representative members of the S100 family, including S100B, S100A1, and S100A9 (Fig. 4b-d, Figure S29) were used as model proteins. Each protein was initially denatured and subsequently incubated with trypsin overnight to ensure complete proteolysis. The predicted peptide sequences of the enzymatic digestion fragments are listed in Table S5. The resulting digested mixture was then filtered to remove residual trypsin, and the peptide fragments were added to *cis* compartment for measurement. As expected, characteristic signals corresponding to peptide fragments of each protein were observed (Fig. 4e-g), regardless of the minor background events present in the blank control (trypsin alone, Figure S30). Events acquired from a 30-min continuous recording were analyzed using a k-means clustering algorithm based on the standard deviation and *ΔI*_*b*_*/I*_*0*_. This analysis revealed distinct distribution patterns for the three proteins, enabling clear differentiation among S100B, S100A1, and S100A9 (Fig. 4h–j). The reproducibility of these clustering patterns was further confirmed by three independent experiments for each condition (Figure S31).

**Fig. 5:**
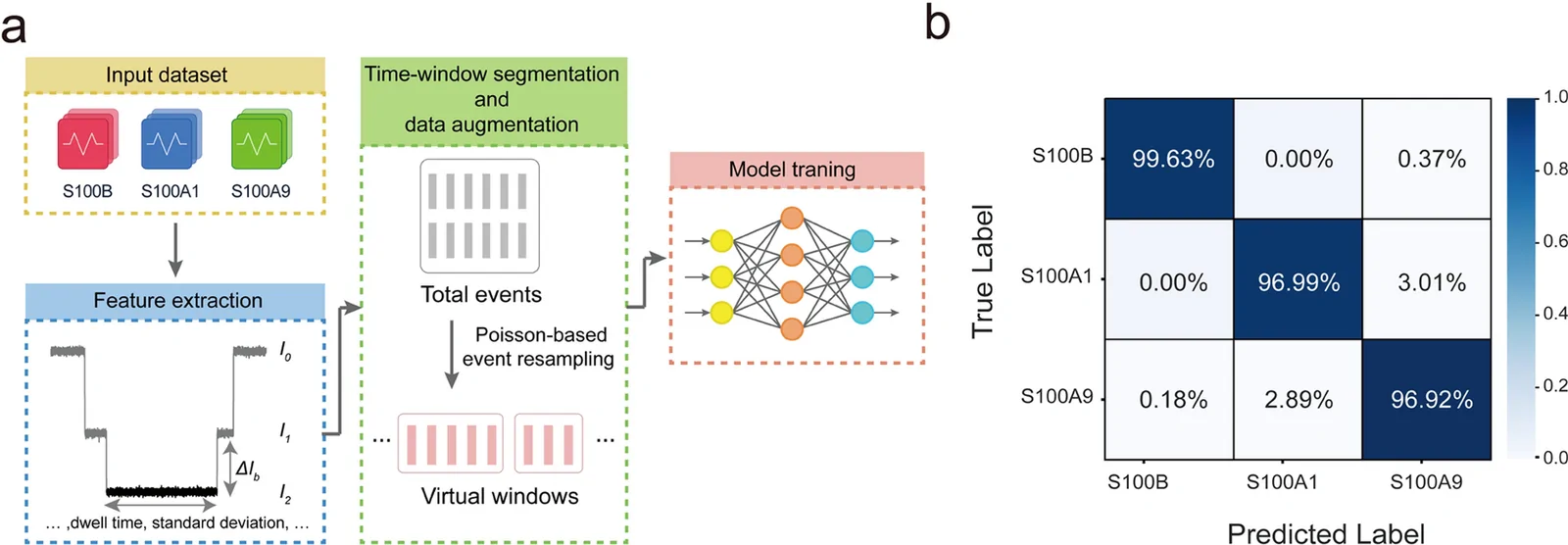
Deep-learning-assisted protein identification from nanopore fingerprints of proteolytic peptide fragments. (**a**) Fifteen-dimensional feature vectors were first extracted from nanopore translocation events generated by enzymatically digested protein samples. Events occurring within an 80 s observation window were then grouped as unordered event sets for holistic protein-level classification. A Poisson-distribution-based event resampling strategy was applied for data augmentation, and model performance was evaluated using leave-one-pore-out cross-validation to minimize pore-dependent bias. (**b**) The confusion matrix of the Deep Sets model for classification of S100B, S100A1, and S100A9 under 15-fold data augmentation. The model achieved high classification accuracy across the three proteins, with an average accuracy of 97.85%

To further improve the automatic identification of different proteins, a deep-learning algorithm was established (Fig. 5a). Specifically, we proposed a time-window-based Deep Sets classification algorithm. This method extracts 15-dimensional comprehensive features (including *ΔI*_*b*_*/I*_*0*_, dwell time, normalized mean current (*ΔI*_*mean*_*/I*_*0*_), normalized median current (*ΔI*_*median*_*/I*_*0*_), normalized standard deviation, normalized variance, skewness, kurtosis, normalized 25th percentile, normalized 75th percentile, normalized interquartile range, normalized minimum current, normalized maximum current, normalized current range, and coefficient of variation) from the translocation events of enzymatically digested peptides, and aggregates the events within an 80-s observation window into an unordered set for holistic classification. To eliminate background interference caused by pore-to-pore variations, the experiments strictly employed a Leave-One-Pore-Out Cross-Validation (LOPO-CV) strategy. Furthermore, we introduced a Poisson-distribution-based event resampling data augmentation strategy to expand the effective sample size. The results demonstrate that under a 15-fold data augmentation, the proposed Deep Sets model achieves an average accuracy of 97.85% (Fig. 5b). This automated framework supports protein identification based on nanopore fingerprints of proteolytically generated peptide fragments with quantified classification performance, providing a complementary strategy to previous nanopore peptide-profiling studies that relied on similarity-based spectral matching for protein identification [15].

By analyzing proteolytically generated peptide fragments, our am8γCD-assisted nanopore strategy shows promising potential for protein identification and characterization. Recent studies using solid-state nanopores have also demonstrated robust strategies for protein fingerprinting. For example, Soni et al. reported cysteine-based protein fingerprinting using solid-state nanopores [64], and Wang et al. developed a supercharged polypeptide carrier-assisted strategy for nanopore protein detection [65]. While these solid-state nanopore strategies provide valuable fingerprinting information at the protein level, our fragmentation-based approach establishes an alternative peptide-level fingerprinting strategy by generating fragment-specific signatures.

## Conclusion

In this study, we proposed an am_8_γCD-assisted strategy based on an arginine-engineered nanopore for highly sensitive analysis of short peptides, wherein am_8_γCD serves as a molecular adaptor that hosts the peptide and enhances detection resolution during translocation. The nanopore-am_8_γCD sensor enables efficient discrimination of uniformly charged homopeptides with single-amino acid resolution, providing a well-defined model system for peptide analysis. Compared with previously nanopore studies, our peptide sensing strategy enables label-free detection of peptides as short as 2 amino acids with a well-resolved dwell time of ~ 3.72 ms (Table S6), highlighting its advantage for ultrashort peptide analysis. Building on this capability, the nanopore sensing system further demonstrated robust sensitivity towards to three representative PTMs in peptides, including acetylation, lactylation, and phosphorylation. Under appropriate experimental conditions, this approach could be expanded to detect a broader range of PTMs. Furthermore, we extended this platform to the analysis of proteolytically generated peptide fragments, thereby establishing a nanopore-based strategy for accurate protein identification. Similar to other protein fingerprinting methods, the broader application of this strategy will benefit from databases of nanopore-based protein fragment fingerprints and robust deep-learning models. In the future, this approach may be further extended to complex biofluids and integrated with high-throughput nanopore platforms for point-of-care protein identification.

### Supporting information

The Supporting Information is available free of charge.

Purification and characterization of α-HL nanopores and S100 proteins; characterization of short peptides; nanopore sensing events of short peptides and digestion products of S100 proteins; mean blockade currents of peptides E_n_ and D_n_.

## Supplementary Information

Supplementary material 1.

## Data Availability

All data supporting the findings of this study are available within the paper and its Supplementary Information.

## Abbreviations

PTMs: Post-translational modifications
MS: Mass spectrometry
α-HL: α-Hemolysin
NaCl: Sodium chloride
KCl: Potassium chloride
IPTG: Isopropyl *β*-D-1-thiogalactopyranoside
HEPES: 4-(2-hydroxyethyl)-1-piperazineethanesulfonic acid
DPhPC: 1, 2-Diphytanoyl-sn-glycero-3-phosphocholine
DTT: DL-Dithiothreitol
GdnHCl: Guanidine hydrochloride
am8γCD: Octakis-(6-amino-6-deoxy)-γ-cyclodextrin
En: Glutamate homopeptides
α-HL WT: Wild-type α-HL nanopore
*I*_*p*_: Mean blockade current
DBSCAN: Density-based spatial clustering of applications with noise
Dn: Aspartate homopeptides
