# Supplementary material for: Discriminating single-amino acid differences and post-translational modifications in negatively charged peptides with α-hemolysin nanopores
Source: J Nanobiotechnology. 2026 Jul 21;24:957. doi: 10.1186/s12951-026-04818-z (PMC13670695; doi:10.1186/s12951-026-04818-z)
Supplement: Supplementary file 1 [file 12951_2026_4818_MOESM1_ESM.docx]

Supplementary Information

**Discriminating Single-Amino Acid Differences and Post-Translational Modifications in Peptides with α-Hemolysin Nanopores**

Liting Kang^1,2^, Linting Wang^2^, Xuefei Fan^2^, Lingfang Xu^2^, Han Yang^2^, Chenxin

Wu^2^, Tongxin Zhu^2^, Xiangyi Zhao^2^, Linming Zhang^3^, Jianhuang Xue^1,*^, Shuanghong Yan^1, 2,*^

1 Key Laboratory of Spine and Spinal Cord Injury Repair and Regeneration of Ministry of Education, Tongji Hospital affiliated to Tongji University, Frontier Science Center for Stem Cell Research, School of Life Sciences and Technology, Tongji University, Shanghai 200092, China

2 School of Pharmacy, Hangzhou Normal University, Hangzhou, Zhejiang 311121, China

3 Shuangming Biotechnology (Suzhou) Co., Ltd., Suzhou, Jiangsu 215000, China

^*^Corresponding authors. Emails: 20230006@hznu.edu.cn (S.Y.), xuejianhuang@tongji.edu.cn (J.X.)

**Figure S1. Purification and characterization of α-HL WT nanopore. (a)** UV absorbance spectra recorded from nickel affinity column elution of α-HL WT during chromatography purification. With a gradient elution of imidazole (0-500 mM), non-specific proteins and target α-HL nanopores were eluted sequentially according to their different binding affinities to the nickel affinity column. The identities of the indicated fractions were further confirmed by gel electrophoresis. **(b)** Gel electrophoresis results of the elution fractions shown in **a**, resolved on a 10% polyacrylamide gel. Lane M: protein standard; lane 1-7: the corresponding elution fractions in **a**. The α-HL WT nanopore was confirmed in fraction 5.

**Figure S2. Single channel recordings of the WT α-HL nanopore. (a)** A representative current trace showing sequential insertions of WT α-HL at +60 mV. The nanopore measurements were performed in 1 M KCl, 10 mM Tris at pH 7.5. **(b)** The histogram of open pore current for WT α-HL nanopore acquired as described in **a**. **(c)** A representative I-V curve of an WT α-HL nanopore.

**Figure S3.** **Sensitivity of the α-HL WT nanopore for peptide detection.** Representative current traces of the α-HL WT nanopore in the absence **(a)** and presence **(b)** of am_8_γCD. Representative current traces of glutamate homopeptide E_4_ translocation through the α-HL WT nanopore in the absence **(c)** and presence **(d)** of am_8_γCD. No detectable translocation events were observed without am_8_γCD, and only transient spiky events were recorded in its presence.

**Figure S4. Schematic illustration of the α-HL (M113R/T145R) nanopore, with the M113R and T145R mutation sites highlighted.**

**Figure S5. Purification and characterization of α-HL (M113R/T145R) nanopore. (a)** UV absorbance spectra recorded from nickel affinity column elution of α-HL (M113R/T145R) during chromatography purification. With a gradient elution of imidazole (0-500 mM), non-specific proteins and target α-HL nanopores were eluted sequentially according to their different binding affinities to the nickel affinity column. The identities of the indicated fractions were further confirmed by gel electrophoresis. **(b)** Gel electrophoresis results of the elution fractions shown in **a**, resolved on a 10% polyacrylamide gel. Lane M: protein standard; lane 1-6: the corresponding elution fractions in **a**. The α-HL (M113R/T145R) nanopore was confirmed in fraction 5-6.

**Figure S6. Single channel recordings of the α-HL (M113R/T145R) nanopore. (a)** A representative current trace showing sequential insertions of α-HL (M113R/T145R) at +60 mV. The nanopore measurements were performed in 1 M KCl, 10 mM Tris at pH 7.5. **(b)** The histogram of open pore current for α-HL (M113R/T145R) nanopore acquired as described in **a**. **(c)** A representative I-V curve of an α-HL (M113R/T145R) nanopore.

**Figure S7. Schematic illustration of the directions of EPF and EOF during peptide translocation through α-HL (M113R/T145R) nanopores.** Cartoons showing the force directions for negatively charged (a), electrically neutral (b), and positively charged (c) peptides. The red arrows indicate electroosmotic flow (EOF), and the green arrows indicate electrophoretic force (EPF).

**Figure S8.** **Sensitivity of the α-HL (M113R/T145R) nanopore for peptide detection.** Representative current traces of the α-HL (M113R/T145R) nanopore in the absence **(a)** and presence **(b)** of am_8_γCD. Representative current traces of glutamate homopeptide E_4_ translocation through the α-HL (M113R/T145R) nanopore in the absence **(c)** and presence **(d)** of am_8_γCD. Peptide E_4_ produced only transient spiky events for α-HL (M113R/T145R) nanopore in the absence of cyclodextrin adaptor, while it generated distinct blockade current signals for α-HL M113R/T145R nanopore with am_8_γCD.

**Figure S9. Characteristics of peptide E_4_ translocation through the α-HL nanopore. (a-e)** Representative current traces of peptide E_4_ with a +50 mV, +55 mV, +60 mV, +65 mV and +70 mV applied potential, respectively. **(f)** Plots of dwell time as a function of the applied voltages. The statistics of dwell time were obtained from three independent measurements, each with a duration of 20 minutes under the respective condition.

**Figure S10. Molecular docking analysis of the peptide with the engineered nanopore-cyclodextrin sensing systems.** Docking models of peptide E4 with α-HL (M113R/T145R)-am8γCD sensor **(a)** and α-HL (M113R/T145R)-γCD sensor **(b)**. E4 is shown with green carbon atoms, cyclodextrin with gray carbon atoms, and the inner-wall residues of the nanopore with blue carbon atoms. Hydrogen bonds and salt bridges are indicated by yellow and red dashed lines, respectively, with interaction distances labeled in Å.

**Figure S11. ESI-MS analysis of the glutamate homopeptides of different lengths. (a)** ESI-mass spectrum of peptide E_2_. The theoretical mass was calculated to be 276.24 Da, which matches the observed mass of 276.24 Da. **(b)** ESI-mass spectrum of peptide E_3_. The theoretical mass was calculated to be 405.36 Da, which matches the observed mass of 405.39 Da. **(c)** ESI-mass spectrum of peptide E_4_. The theoretical mass was calculated to be 534.47 Da, which matches the observed mass of 534.53 Da. **(d)** ESI-mass spectrum of peptide E_5_. The theoretical mass was calculated to be 663.59 Da, which matches the observed mass of 663.62 Da. **(e)** ESI-mass spectrum of peptide E_6_. The theoretical mass was calculated to be 792.70 Da, which matches the observed mass of 792.35 Da. **(f)** ESI-mass spectrum of peptide E_7_. The theoretical mass was calculated to be 921.81 Da, which matches the observed mass of 921.62 Da. **(g)** ESI-mass spectrum of peptide E_8_. The theoretical mass was calculated to be 1051.93 Da, which matches the observed mass of 1050.20 Da. **(h)** ESI-mass spectrum of peptide E_9_. The theoretical mass was calculated to be 1180.04 Da, which matches the observed mass of 1180.20 Da. **(i)** ESI-mass spectrum of peptide E_10_. The theoretical mass was calculated to be 1309.16 Da, which matches the observed mass of 1309.20 Da.

**Figure S12. Dwell-time distributions of glutamate homopeptides E_4_–E_10_ in nanopore sensing.** Dwell-time histograms for E_4_ **(a)**, E_5_ **(b)**, E_6_ **(c)**, E_7_ **(d)**, E_8_ **(e)**, E_9_ **(f)**, E_10_ **(g)**.

**Figure S13. Event statistics derivation.** **(a)** A representative current trace of peptide sensing. E_4_ was employed as a model analyte. *I_0_* represents the open pore current. *I_1_* represents the blockade level generated by am_8_γCD bound within the α-HL (M113R/T145R) nanopore. *I_2_* represents the blockade current generated by peptide binding to the nanopore-CD complex. The event dwell time and *ΔI_b_* were defined as marked in the current trace. **(b)** Event histogram of *ΔI_b_/I_0_* for E_4_. Event histogram of *ΔI_b_/I_0_* for E_4_. The peak in the histogram was fitted using a Gaussian function defined as: $y=y_{0}+Aexp(-\frac{\left( x-x_{c} \right)^{2}}{2w^{2}})$. The resulting peak value *x_c_* is used as the mean blockade current (*I_p_*). The measurement was performed as described in **Methods**. E_4_ was added to the *cis* chamber to a final concentration of 0.4 μM. A constant voltage of +60 mV was applied throughout the measurements.

**Figure S14. A representative trace acquired during simultaneous sensing of seven glutamate homopeptides (E_4_-E_10_).** Events from different peptides were recognized from the trace and marked with corresponding color bars. Different glutamate homopeptides were simultaneously added to the *cis* compartment, each at a final concentration of 0.4 μM. A constant voltage of +60 mV was continually applied during the measurement.

**Figure S15. Detection of peptides E_2_ and E_3_.** Representative current traces of E_2_ (**a**) and E_3_ (**b**) sensing by an α-HL (M113R/T145R) nanopore equipped with am_8_γCD. The current traces show detectable blockade events of peptides E_2_ and E_3_. The measurements were carried out in an aqueous buffer of 0.5 M KCl, 10 mM Tris, pH 7.5. A constant voltage of +70 mV was applied throughout the measurements. The peptide was added to the *cis* chamber at a final concentration of 1.5 μM. Histograms of *ΔI_b_/I_0_* values of E_2_ **(c)** and E_3_ **(d)**. Dwell-time distributions of E_2_ **(e)** and E_3_ **(f)** in nanopore sensing.

**Figure S16. ESI-MS analysis of the aspartate homopeptides of different lengths.** **(a)** ESI-mass spectrum of peptide D_4_. The theoretical mass was calculated to be 478.36 Da, which matches the observed mass of 478.10 Da. **(b)** ESI-mass spectrum of peptide D_5_. The theoretical mass was calculated to be 593.45 Da, which matches the observed mass of 593.25 Da. **(c)** ESI-mass spectrum of peptide D_6._ The theoretical mass was calculated to be 708.54 Da, which matches the observed mass of 708.20 Da. **(d)** ESI-mass spectrum of peptide D_7_. The theoretical mass was calculated to be 823.63 Da, which matches the observed mass of 823.40 Da. **(e)** ESI-mass spectrum of peptide D_8_. The theoretical mass was calculated to be 938.71 Da, which matches the observed mass of 938.50 Da. **(f)** ESI-mass spectrum of peptide D_9_. The theoretical mass was calculated to be 1053.80 Da, which matches the observed mass of 1053.60 Da. **(g)** ESI-mass spectrum of peptide D_10_. The theoretical mass was calculated to be 1168.89 Da, which matches the observed mass of 1168.20 Da.

**Figure S17. Representative nanopore events acquired during simultaneous measurement of seven aspartate homopeptides (D_4_ to D_10_).** Seven distinct blockade events were generated by different peptides. The experiment was performed in the buffer of 1 M KCl, 10 mM Tris at pH 7.5. Each peptide was added to the *cis* compartment at a final concentration of 0.4 μM.

**Figure S18. Statistics of *I_p_* acquired with aspartate homopeptides D_4_ to D_10_.** Summary of the mean and standard deviation of *I_p_* (mean blockade current) measured with various different aspartate homopeptides. The experiments were carried out in an aqueous buffer containing 1 M KCl, 10 mM Tris at pH 7.5. A voltage of +60 mV was continually applied during the measurements. Three independent measurements were performed for each peptide to obtain the statistics.

**Figure S19. Dwell-time distributions of aspartate homopeptides D4–D10 in nanopore sensing.** Dwell-time histograms for D_4_ **(a)**, D_5_ **(b)**, D_6_ **(c)**, D_7_ **(d)**, D_8_ **(e)**, D_9_ **(f)**, D_10_ **(g)**.

**Figure S20. ESI-MS analysis of peptides W_10_, G_10_ and K_10_.** **(a)** ESI-mass spectrum of peptide W_10_. The theoretical mass was calculated to be 1880.11 Da, which matches the observed mass of 1880.86 Da. **(b)** ESI-mass spectrum of peptide G_10_. The theoretical mass was calculated to be 588.53 Da, which matches the observed mass of 588.67 Da. **(c)** ESI-mass spectrum of peptide K_10_. The theoretical mass was calculated to be 1299.74 Da, which matches the observed mass of 1300.02 Da.

**Figure S21. Detection of peptides W_10_, G_10_, K_10_.** Representative current traces of W_10_ **(a)**, G_10_ **(b)** and K_10_ **(c)** sensing by α-HL (M113R/T145R)-am_8_γCD sensors. The current traces show detectable blockade events of peptides W_10_, G_10_, K_10_. The measurements were carried out in an aqueous buffer of 1 M KCl, 10 mM Tris, pH 7.5. Peptides W_10_ and G_10_ were added individually to the *cis* compartment at final concentrations of 50 μM and 100 μM, respectively. Peptide K_10_ was added to the *trans* compartment at a final concentration of 20 μM. A +60 mV voltage was continually applied during the measurements. Histograms of *ΔI_b_/I_0_* values of W_10_ **(d),** G_10_ **(e)** and K_10_ **(f)**.

**Figure S22. Event histogram of *ΔI_b_/I_0_* for the thermally treated D_8_ sample.** After treatment at 100 °C for 1 h, the D_8_ degradation mixture showed five major event populations, corresponding to D_4_, D_5_, D_6_, D_7_, and intact D_8_, respectively.

**Figure S23. Mass spectrometry analysis of the thermally treated D_8_ sample.** The mass spectrum confirmed the presence of D_4_, D_5_, D_6_, D_7_, and intact D_8_ species in the same degradation mixture analyzed by nanopore sensing in **Figure S22**.

**Figure S24. Nanopore analysis of bivalirudin.** Representative current traces of untreated bivalirudin **(a)** and bivalirudin thermally treated at 100 °C for 3 h **(b)**, respectively. Two types of events were observed: type 1 events originated from the open-pore current level, whereas type 2 events appeared as further blockades from the am8γCD-associated current level. Untreated bivalirudin mainly produced type 1 events, likely due to limited size compatibility between the 20-amino-acid peptide and the cyclodextrin adaptor. Numbers of type 1 and type 2 events recorded during a 10 min nanopore measurement for untreated **(c)** and thermally treated bivalirudin **(d)**, respectively. Event histograms of *ΔI_b_/I_0_* for type 1 **(e)** and type 2 events **(g)** of untreated bivalirudin. Event histograms of *ΔI_b_/I_0_* for type 1 **(f)** and type 2 events **(h)** of thermally treated bivalirudin, respectively.

**Figure S25. Mass spectrum of thermally treated bivalirudin.** Bivalirudin was thermally treated at 100 °C for 3 h before mass spectrometry analysis. The peak corresponding to intact bivalirudin (2178.99 Da) was no longer detectable after thermal treatment, confirming bivalirudin degradation.

**Figure S26. ESI-MS analysis of peptides KD_4_S, (KD_4_S)-Ac, (KD_4_S)-La and (KD_4_S)-P.** **(a)** ESI-mass spectrum of KD_4_S. The theoretical mass was calculated to be 693.61 Da, which matches the observed mass of 693.62 Da. **(b)** ESI-mass spectrum of peptide (KD_4_S)-Ac. The theoretical mass was calculated to be 735.62 Da, which matches the observed mass of 735.84 Da. **(c)** ESI-mass spectrum of peptide (KD_4_S)-La. The theoretical mass was calculated to be 765.68 Da, which matches the observed mass of 766.72 Da. **(d)** ESI-mass spectrum of peptide (KD_4_S)-P. The theoretical mass was calculated to be 773.60 Da, which matches the observed mass of 773.78 Da.

**Figure S27. Scatter plots of standard deviation versus ΔI_b_/I_0_ of events from peptides KD_4_S, (KD_4_S)-Ac, (KD_4_S)-La and (KD_4_S)-P.** Scatter plots of standard deviation versus *ΔI_b_/I_0_* of peptides KD_4_S **(a)**, (KD_4_S)-Ac **(b)**, (KD_4_S)-La **(c)** and (KD_4_S)-P **(d)** for three independent measurements were demonstrated. All acquired events were processed by cluster analysis using DBSCAN to remove noise events. For the analysis of KD_4_S, (KD_4_S)-Ac, and (KD_4_S)-La, the DBSCAN parameters were set as epsilon=0.05 and min_samples=10. For the analysis of (KD_4_S)-P, the DBSCAN parameters were set as epsilon=0.05 and min_samples=15. The experiments were carried out in an aqueous buffer containing 1 M KCl, 10 mM Tris at pH 7.5. The peptide was added to the *cis* compartment with a final concentration of 0.4 μM. A voltage of +60 mV was continually applied during the measurement.

**Figure S28. Quantitative determination of the molar ratio of (KD4S)-P and KD4S in mixed samples.** **(a)** Calibration curve correlating the nanopore signal count ratio of (KD4S)-P to KD4S with the corresponding molar ratio in mixed peptide samples. The linear fit shows a good correlation between the signal count ratio and molar ratio. **(b)** Comparison between the prepared molar ratios (addition) and the molar ratios calculated from nanopore detection (detection) for four independently prepared mixed samples.

**Figure S29. Purification and characterization of S100B, S100A1 and S100A9 proteins.** UV absorbance spectra recorded from nickel affinity column elution of S100B **(a)**, S100A1 **(c)** and S100A9 **(e)** during chromatography purification. With a gradient elution of imidazole (0-500 mM), non-specific proteins and target proteins were eluted sequentially according to their differing binding affinities to the nickel affinity column. The identities of the indicated fractions were further confirmed by gel electrophoresis. **(b)** Gel electrophoresis results for different elution fractions in **a**. Lane M: protein standard; lane 1-4: the corresponding elution fractions in **a**. The S100B protein was confirmed in fraction 3. **(d)** Gel electrophoresis results for different elution fractions in **c**. Lane M: protein standard; lane 1-7: the corresponding elution fractions in **c**. The S100A1 protein was confirmed in fraction 6. **(f)** Gel electrophoresis results for different elution fractions in **e**. Lane M: protein standard; lane 1-10: the corresponding elution fractions in **e**. The S100A9 protein was confirmed in fraction 7-10. The elution fractions were analyzed by 10% polyacrylamide gel electrophoresis.

**Figure S30.** **Blank control experiment showing nanopore signals from trypsin autolysis.** **(a)** Representative current trace of the trypsin-only control after overnight incubation under the same conditions as the protein samples. **(b)** Histogram of *ΔI_b_/I_0_* values of trypsin-only control generated from 30 min continuous recording. A voltage of +60 mV was continually applied during the measurements.

**Figure S31. Reproducibility of proteolytic peptide fragment detection.** Scatter plots of the standard deviation versus *ΔI_b_/I_0_* for nanopore events corresponding to the digestion products of S100B **(a)**, S100A1 **(b)** and S100A9 **(c)** across three independent measurements. The measurements were carried out as described in **Methods**. All the scatter plots were respectively generated from 30 min continuous recording. The digestion products were added to the *cis* compartments to reach a final concentration of 20 ng/μL. A voltage of +60 mV was continually applied during the measurements.

**Table S1.**

**Peptides sequences in this study.**

| **Name** | **Sequence** |
| --- | --- |
| E_2_ | EE |
| E_3_ | EEE |
| E_4_ | EEEE |
| E_5_ | EEEEE |
| E_6_ | EEEEEE |
| E_7_ | EEEEEEE |
| E_8_ | EEEEEEEE |
| E_9_ | EEEEEEEEE |
| E_10_ | EEEEEEEEEE |
| D_4_ | DDDD |
| D_5_ | DDDDD |
| D_6_ | DDDDDD |
| D_7_ | DDDDDDD |
| D_8_ | DDDDDDDD |
| D_9_ | DDDDDDDDD |
| D_10_ | DDDDDDDDDD |
| bivalirudin | FPRPGGGGNGDFEEIPEEYL |
| KD_4_S | KDDDDS |
| (KD_4_S)-Ac | K(Ac)DDDDS |
| (KD_4_S)-La | K(La)DDDDS |
| (KD_4_S)-P | KDDDDS(P) |
| W_10_ | WWWWWWWWWW |
| G_10_ | GGGGGGGGGG |
| K_10_ | KKKKKKKKKK |

**Table S2.**

**Molecular docking data of peptide E4 with the α-HL (M113R/T145R)-am8γCD sensor and α-HL (M113R/T145R)-γCD sensor.**

| **Docking System** | **Affinity Score (kcal/mol)** | **Hydrogen Bond Interaction** | **Hydrogen Bond Interaction** |
| --- | --- | --- | --- |
| E_4_+α-HL (M113R/T145R)-am_8_γCD | -7.0 | 15 | 10 |
| E_4_+α-HL (M113R/T145R)-γCD | -6.5 | 8 | 7 |

**Table S3.**

**Summary of the mean and standard deviation of** ***I_p_* (Figure S5) for different homopeptides**. All measurements were conducted as described in SI method. The peptide was added to the *cis* compartment at a final concentration of 0.4 µM. A voltage of +60 mV was continuously applied during the measurement. All statistical results were derived from three independent measurements.

| Name | *I_p_* | | | | Mean±SD |
| --- | --- | --- | --- | --- | --- |
|  | #1 | #2 | #3 | |  |
| E_4_ | 0.232 | 0.233 | 0.229 | 0.231±0.002 | |
| E_5_ | 0.285 | 0.280 | 0.279 | 0.281±0.003 | |
| E_6_ | 0.324 | 0.320 | 0.319 | 0.321±0.003 | |
| E_7_ | 0.353 | 0.346 | 0.345 | 0.348±0.004 | |
| E_8_ | 0.378 | 0.372 | 0.369 | 0.373±0.005 | |
| E_9_ | 0.389 | 0.388 | 0.387 | 0.388±0.001 | |
| E_10_ | 0.406 | 0.399 | 0.399 | 0.401±0.004 | |
| D_4_ | 0.229 | 0.237 | 0.229 | 0.232±0.005 | |
| D_5_ | 0.278 | 0.285 | 0.280 | 0.281±0.004 | |
| D_6_ | 0.313 | 0.325 | 0.316 | 0.318±0.006 | |
| D_7_ | 0.338 | 0.349 | 0.337 | 0.341±0.007 | |
| D_8_ | 0.362 | 0.361 | 0.359 | 0.361±0.002 | |
| D_9_ | 0.378 | 0.378 | 0.375 | 0.377±0.002 | |
| D_10_ | 0.387 | 0.393 | 0.391 | 0.390±0.003 | |

**Table S4.** **Comparison with the previous FraC nanopore-based peptide detection method.**

| **Feature** | ***Nat Commun* 10, 835 (2019)** | **Our study** |
| --- | --- | --- |
| Nanopore system | FraC nanopores | α-HL (M113R/T145R)+ am_8_γCD |
| Detectable peptide length | 4-22 aa | 2-10 aa |
| Experimental condition | Multiple FraC pore types, under different voltage conditions | α-HL (M113R/T145R)  under the same experimental conditions |
| Minimum peptide length | 4 aa | 2 aa |
| Dwell time for 4-aa peptide | ~0.32 ms | ~3.03 ms |
| PTM compatibility | Not explicitly addressed | Compatible under the same conditions |

**Table S5. Peptide sequences of enzymatic digestion fragments from S100 proteins.**

| **S100B** | | |
| --- | --- | --- |
| **Sequence** | **Length** | **Mass (Da)** |
| K | 1 | 128.17 |
| LK | 2 | 259.35 |
| HK | 2 | 283.33 |
| EGDK | 4 | 447.45 |
| SELK | 4 | 475.26 |
| MSELEK | 6 | 627.77 |
| EQEVVDK | 7 | 845.91 |
| ELINNELSHFLEEIK | 15 | 1828.02 |
| AMVALIDVFHQYSGR | 15 | 1455.65 |
| VMETLDNDGDGECDFQEFMAFVAMVTTACHEFFEHELEHHHHHH | 44 | 4513.09 |
| **S100A1** | | |
| **Sequence** | **Length** | **Mass (Da)** |
| K | 1 | 128.17 |
| YK | 2 | 327.38 |
| VMK | 3 | 376.51 |
| LSK | 3 | 346.43 |
| ELK | 3 | 388.46 |
| DADAVDK | 7 | 732.75 |
| ELDENGDGEVDFK | 13 | 1466.47 |
| DLLQTELSGFLDVQK | 15 | 1705.94 |
| MGSELESAMETLINVFHAHSGQEGDK | 26 | 2818.08 |
| EYVVLVAALTVACNNFFWETSHHHHHH | 27 | 3354.71 |
| **S100A9** | | |
| **Sequence** | **Length** | **Mass (Da)** |
| K | 1 | 128.17 |
| QK | 2 | 274.32 |
| EMK | 3 | 406.50 |
| QLVK | 4 | 486.61 |
| LNQK | 4 | 501.58 |
| LEHPDK | 6 | 737.82 |
| ELPNFLK | 7 | 860.02 |
| NDNAINK | 7 | 805.83 |
| IMEDLDTNGDK | 11 | 1268.36 |
| LTVASHEEMHK | 11 | 1281.46 |
| ELNFEEFSILVAR | 13 | 1584.81 |
| MADQMSQLECSIETIINIFHQYSVR | 25 | 3047.46 |
| NAPEGEGHSHGPGFGEGSQGHCHSHGGHGHGHSHHHHHH | 39 | 4222.21 |

**Table S6. Comparison of nanopore-based studies of peptide detection.**

| **Strategy** | **Representative system** | **Sample labeling required** | **Shortest peptide length reported** | **reported dwell time/translocation speed** | **reference** |
| --- | --- | --- | --- | --- | --- |
| Intrinsic peptide trapping | WT aerolysin | No | 5 aa | 0.9 ms | *Nat Commun* 9, 966 (2018). |
| Pore-size engineering | FraC nanopore | No | 4 aa | 0.32 ms | *Nat Commun* 10, 835 (2019) |
| EOF-enhanced peptide sensing | T232K aerolysin nanopore | No | 5 aa | ms-level; no separate mean dwell time reported for the 5-aa peptide | *Nat. Chem.* 15, 578–586 (2023) |
| Enzyme-mediated strategy | MspA nanopore+phi29 DNAP | Yes | 25 aa | Stepwise movement observed; explicit peptide translocation speed not reported | *Nano Lett.* 21, 6703–6710 (2021) |
| **Cyclodextrin-assisted sensing with engineered α-HL** | **α-HL (M113R/T145R) + am_8_γCD** | **No** | **2 aa** | **~3.72 ms** | **Our study** |
